# Systematic analysis of MCM3 in pediatric medulloblastoma *via* multi-omics analysis

**DOI:** 10.3389/fmolb.2022.815260

**Published:** 2022-09-05

**Authors:** Liangliang Cao, Yang Zhao, Zhuangzhuang Liang, Jian Yang, Jiajia Wang, Shuaiwei Tian, Qinhua Wang, Baocheng Wang, Heng Zhao, Feng Jiang, Jie Ma

**Affiliations:** Department of Pediatric Neurosurgery, Xin Hua Hospital Affiliated to Shanghai Jiao Tong University School of Medicine, Shanghai, China

**Keywords:** MCM3, medulloblastoma, multi-omics, DNA methylation, single cell, RNA sequencing, prognosis, nomogram

## Abstract

Minichromosome maintenance proteins are DNA-dependent ATPases that bind to replication origins and allow a single round of DNA replication. One member of this family, MCM3, is reportedly active in most cancers. To systematically elucidate the mechanisms affected by aberrant MCM3 expression and evaluate its clinical significance, we analyzed multi-omics data from the GEO database and validated them in cell lines and tumor samples. First, we showed the upregulation of MCM3 in medulloblastoma (MB) at bulk and single-cell RNA sequence levels and revealed the potential role of MCM3 *via* DNA replication. Then we found the dysregulation of MCM3 might result from abnormal methylation of MCM3. Moreover, we discovered that MCM3 might affect varied biological processes such as apoptosis, autophagy, and ferroptosis and that MCM3 was correlated with immune components such as fibroblast and neutrophils, which were associated with overall survival in different medulloblastoma subtypes. Furthermore, we found that MCM3 expression was correlated with the IC_50_ values of cisplatin and etoposide. The nomogram of MCM3-related genes showed the reliable and better prediction of 1- and 5-year survival compared to current histological and molecular classifications. Overall, the results of our study demonstrated that MCM3 might serve as a potential biomarker with clinical significance and better guidance than current histological and molecular classifications for clinical decision-making.

## Introduction

Medulloblastoma (MB), the leading cause of cancer-related death in children, is one of the most common pediatric brain tumors (Hovestadt et al., 2020). In recent years, individualized therapy models have emerged based on molecular subtypes and risk stratification. Surgical resection, cytotoxic chemotherapy, and craniospinal irradiation (for non-infants usually ≥3 years of age at diagnosis) constitute the standard therapy for MB. The estimated 5-year overall survival has remained unchanged during the past two decades, ranging from 60% to 80% (Lannering et al., 2012; von Bueren et al., 2016). Despite these high estimates, the drawbacks of current treatment strategies include toxic effects on neurocognition and the neuroendocrine systems, sluggish identical therapies concerning radiotherapy and cytotoxic chemotherapy in developing children, lack of indicators for novel clinical medications, etc. Therefore, more therapeutic targets and less toxic strategies are required.

Non-invasive methods have made great advances with the identification of molecular subtypes based on DNA methylation. Similarly, as another clinical evaluation method, magnetic resonance imaging (MRI) also helps the differentiation of medulloblastoma from other pediatric brain tumors and risk stratification based on different features of T1 and T2-weighted MRI (Duc et al., 2020; Minh Thong and Minh Duc, 2020; Zhang et al., 2022). In addition, cellular proliferation plays an essential role in tumor content, especially in highly malignant cancers (Gu et al., 2021; McCarthy et al., 2021; Newman et al., 2021; Qiu et al., 2021). As the molecular mechanisms involved have been uncovered gradually, increasing numbers of informative biomarkers have been identified to evaluate the degree of malignancy of various cancers, including proliferating cell nuclear antigen (PCNA) and marker of proliferation Ki-67 (MKi67). Additionally, eukaryotic DNA replication guarantees genome stability. The minichromosome maintenance (MCM) proteins play a role as subunits of pre-replication complexes in the G1 phase and bind to replication origins and restrict DNA synthesis to a single round of DNA replication (Madine et al., 1995; Sedlackova et al., 2020). MCM proteins can reflect the cell cycle status due to their stable state during the cell cycle and proteolysis in quiescent cells (G0) (Musahl et al., 1998; Madine et al., 2000). Some studies have reported other functions of MCM proteins in different cancers, such as their relationships to the immune response in brain gliomas (Söling et al., 2005), execution of apoptosis (Schwab et al., 1998), regulation of autophagy (Puustinen et al., 2020), resistance to anti-tumor therapies (Shrestha et al., 2021), and stemness of cancer cells (Wang et al., 2020). The dysregulated expression of MCM3 has also been demonstrated in varied tumors and could serve as a target or prognostic biomarker (Stewart et al., 2017; Iglesias-Gato et al., 2018; Zhao et al., 2020). To date, there is only one study has reported the expression of MCM3 in various MB cell lines, and evidence of its systematic roles in MB remains deficient.

The present study systematically analyzed the functions of MCM3 in pediatric MB combined with clinical tumor specimens *via* multi-mics bioinformatic analysis. The results revealed its potential roles as a therapeutic target and a tool for better guidance compared to current histological and molecular classifications for clinical decision-making.

## Materials and methods

### Public data collection and construction of the validation cohort

The normalized pediatric MB datasets of DNA Methylation, mRNA array, and single-cell RNA sequencing (including GSE85212, GSE54880, GSE85217, GSE42656, GSE50161, and GSE155446) were obtained from Gene Expression Omnibus (GEO, http://www.ncbi.nlm.nih.gov/geo/) database. In addition, 62 clinical tumor tissues were collected from children diagnosed with primary MB who received surgical treatment in our medical center (Xinhua Hospital Affiliated to Shanghai Jiao Tong University of Medicine) between July 2012 and October 2017. The study protocol was approved by the Ethics Committee of the Xinhua Hospital Affiliated with Shanghai Jiao Tong University School of Medicine (Approval No. XHEC-D-2021-076, Approval Date. 2021-10-21). Written informed consents were obtained from all patients.

### RNA sequencing of clinical samples

RNA-seq service was provided by MAJORBIO (Shanghai, China), and completed on a HiSeq4000 instrument. The RNA-seq reads were mapped to the hg38 reference genome using STAR (v2.5.3a) (Dobin et al., 2013). Fragments per kilobase of transcript per million fragments mapped (FPKM) was calculated, and a mean FPKM ≥ 1 was set as the threshold to determine the active genes in all samples.

### Expression features of MCM3 in different cancers

The Gene Expression Profiling Interactive Analysis (GEPIA.2) database was used to determine the differential expression of MCM3 in various cancers (Tang et al., 2019). The expression features in different brain tumors, biological functions, and gene effects in MB cell lines were analyzed from the CCLE database (Ghandi et al., 2019). The function of MCM3 was investigated in the Biological General Repository for Interaction Datasets-Open Repository for CRISPR Screens (BioGRID ORCS) database (Oughtred et al., 2019). The gene effects on cancer cells were studied using the DepMap database (Tsherniak et al., 2017). The relationships between MCM3 and clinical features were investigated with GEO data and validated in our own data by utilizing the R programing language.

### Differential gene expression and functional enrichment analyses

Differential expression analysis was performed with two datasets (GSE42656 AND GSE50161) using R/limma (Ritchie et al., 2015). The differentially expressed genes (DEGs) were obtained by the intersecting DEGs from the two datasets. The DEGs from GSE50161 were analyzed by comparing the tumor group to fetal and adult normal brain tissues, respectively, to reduce the impact of developmental genes. The criteria for DEG analysis were *p* < 0.05 and fold change (FC) > 2. The interaction network between DEGs was predicted using the online STRING tool (Szklarczyk et al., 2021). The hub genes and related biological processes were identified in Cytoscape (ClueGO) (Shannon et al., 2003).

### Data processing and analysis of MCM3 in single-cell RNA sequencing data

The count matrix obtained from GEO was processed using the Seurat package to get the Seurat object, filtered with a criterion of >500 and <7,800 genes, and normalized using the NormalizeData function (Butler et al., 2018). Highly variable genes between cells were then identified using the FindVariableFeatures function for the subsequent principal component analysis (PCA). Ten principal components were presented for uniform manifold approximation and projection (UMAP) dimension reduction to obtain a two-dimensional representation of the cell state. The FindClusters function was used for clustering with a selection of resolution of 0.3. The singleR package was applied for cell annotation, in which non-immune cells were treated as tumor cells for simplified analysis (Aran et al., 2019). The expression values of MCM3 in different clusters or groups were analyzed using the FeaturePlot function in the Seurat package. Cells expressing MCM3 were extracted and classified as showing high or low expression levels according to the mean expression value. Moreover, the DEGs between them were identified using the FindMarkers function and analyzed with enrichment analysis to investigate the biological process affected.

### Effects of MCM3 dysregulation

Single-sample gene set variation analysis (ssGSVA) was performed in R/GSVA to analyze the biological functions between high and low-risk classifications of MCM3 (Sonja et al., 2013). Gene ontology (GO) and pathway enrichment analysis (Kyoto Encyclopedia of Genes and Genomes (KEGG)) were performed in R/clusterProfiler (Yu et al., 2012) to analyze the intersected processes shared between the MCM3-correlated genes and DEGs. *P* values <0.05 and FDR <25% were considered statistically significant. Immune infiltration was evaluated using the “xCell” (Aran et al., 2017), “ESTIMATE” (Yoshihara et al., 2013), and “CIBERSORT” (Newman et al., 2015) packages in R. The immune indexes related to survival rate were identified using the “survival” package in R. Their correlations with MCM3 expression were also analyzed. The genes related to apoptosis, autophagy, and ferroptosis were obtained from corresponding online databases, including Gene Set Enrichment Analysis (GSEA) (Subramanian et al., 2005), AmiGo 2 (Park et al., 2015), HAMdb (Wang et al., 2018) and FerrDb (Zhou and Bao, 2020).

### DNA methylation analysis of MCM3 in MB

Missing values in the beta value matrixes were processed using the “impute” package in R. The probes were filtered and normalized using the “ChAMP” package (Andrew et al., 2014). The data quality was then checked by principle component analysis (PCA) and heatmaps. The MCM3 probes were analyzed via differential, correlation, and survival analyses to identify methylation sites affecting MCM3 expression.

### Prognostic model construction and analysis of drug susceptibility

Survival data of GSE85217 was downloaded and filtered (age <18 years). This discovery set was then randomly divided into training and test sets in a 7:3 ratio. The data set of 62 RNA sequencing data from our medical center was treated as the independent validation set for the prognostic model. The overall survival and MCM3-related genes were identified using univariate Cox regression and lasso regression analyses. A nomogram containing a multigene panel and clinical features was used to predict the survival probability. The pRRophetic package in R was used to predict the drug sensitivity of each sample according to the gene expression matrix and to evaluate the correlation between MCM3 expression and IC_50_ values (Geeleher et al., 2014).

### shRNA plasmid

For the generation of shRNA plasmids, double-strand oligonucleotides were annealed and cloned into the CMV-EGFP-F2A-puro vector. The oligonucleotides of shRNA were synthesized by OBIO Technology (Shanghai, China). The target oligonucleotides were:

shMCM3-1: GGA​TGA​ATC​AGA​GAC​AGA​A; shMCM3-2: GCA​GTC​AAT​CGG​CAT​GAA​T; shMCM3-3: GCC​TCA​CAG​AAT​CCA​TCA​A.

### Cell transfection

D425 and D458 cell lines were kind gifts from Shanghai Jiao Tong University of Medicine School, China. The D283 and D458 cell lines were cultured in Dulbecco’s modified Eagle’s medium (DMEM) supplemented with 1% penicillin–streptomycin and 10% FBS. At 48 h post-infection, the cells were harvested and subjected to protein extraction or other cellular experiments.

### RNA extraction and real-time RT-PCR

Total RNA was extracted using TRIzol Reagent (Takara, 9108) according to the manufacturer’s instructions. A High-capacity cDNA Reverse Transcription Kit (Takara, RRO47A) was used to perform the reverse transcription reactions. Quantitative PCR was performed on an ABI VERTI Real-Time PCR instrument. The relative mRNA levels were normalized to GAPDH. The qPCR primer sequences were:

MCM3, 5′-TCA​GAG​AGA​TTA​CCT​GGA​CTT​CC-3′ (forward); 5′-TCA​GCC​GGT​ATT​GGT​TGT​CAC-3′ (reverse).

### Western blot assay

The target protein was extracted, and its concentration was quantified using a BCA Protein Assay Kit (Pierce, 23227). Protein samples were separated by sodium dodecyl sulfate-polyacrylamide gel (SDS-PAGE) and transferred onto polyvinylidene difluoride (PVDF) membranes (Millipore). The membranes were blocked with 5% fat-free milk (BD Biosciences, 232100) and then incubated with primary antibodies against MCM3 (1:1,000; Cell Signaling Technology, 13421S), MCM2 (1:1,000; Cell Signaling Technology, 12191), MCM7 (1:2,000; Proteintech, 66905-1), and CDC45 (1:1,000; Cell Signaling Technology, 9405S), respectively. The secondary antibodies were HRP-linked goat anti-mouse IgG (1 ml; Cell Signaling Technology, 7076). The chemical fluorescence images of the proteins were visualized using a chemiluminescent substrate (Epizyme Biotech, Shanghai, China).

### Cell proliferation assay

The effects of etoposide (XY91494, X-Y Biotechnology, China) and cisplatin (ST1164, Beyotime, China) on cell proliferation under different conditions were determined using Cell Counting Kit-8 (CKK-8) reagents (B34304, Bimake, China). Cells were plated in 96-well plates (4 replicates per condition), treated with serial drug concentrations, and incubated in normoxic conditions (37°C, 5% CO_2_, 21% O_2_) for 24, 48, and 72 h. The CKK-8 assays were performed to determine the IC_50_ values at each time point.

## Results

### Dysregulated MCM3 expression in pediatric MB and identification of MCM3-related signaling pathways and genes

**FIGURE 1:**
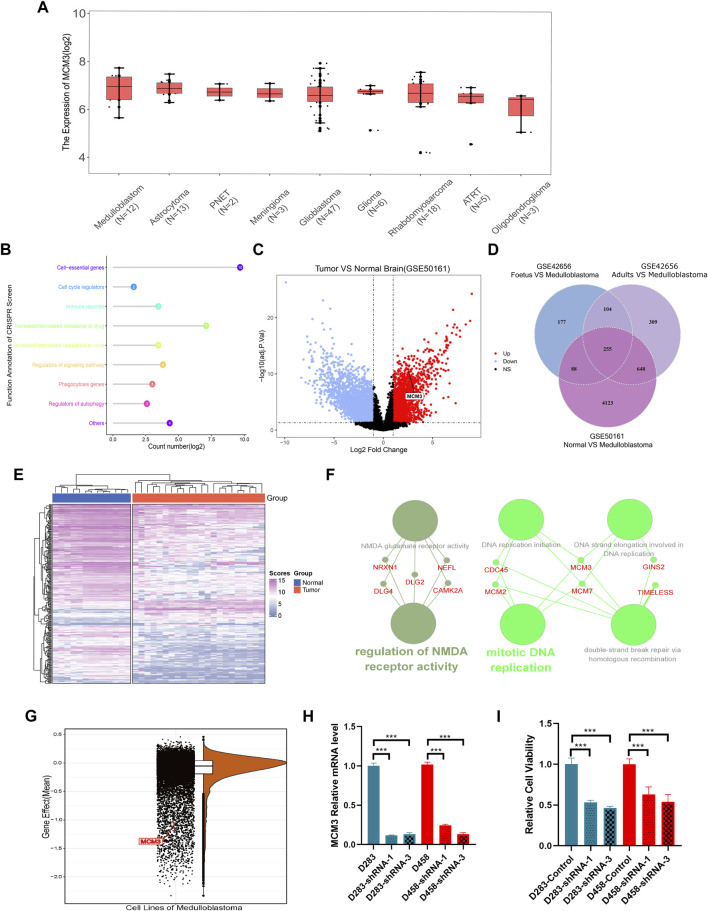
MCM3 expression levels among brain tumor cell lines **(A)**. Functional annotation of MCM3 from the BioGRID ORCS database by CRISPR **(B)**. MCM3 upregulation in the tumor group of GSE50161 **(C)**. 255 DEGs showed dysregulation in the three contrast sets **(D)**. The normal and medulloblastoma samples from GSE50161 clustered respectively according to the expression of DEGs **(E)**. Hub genes analysis *via* Cytoscape showing the main biological functions with which MCM3 is involved **(F)**. Gene effects (namely the dependency of the cell on genes) of MCM3 reflect its essentiality for cancer cell survival **(G)**. Confirmation of MCM3 knockdown by shRNA-1 and shRNA-3 on RNA level by QT-PCR **(H)** in both D283 and D458 cell lines **(H)**. Significantly decreased cell viability in sh-MCM3 cell lines **(I)**.

The RNA-seq data of different brain tumor cell lines from the Cancer Cell Line Encyclopedia (CCLE) showed the highest MCM3 mRNA expression level in MB (Figure 1A). The GEPIA 2 database also revealed higher MCM3 mRNA expression levels in cancer tissues compared to those in normal tissues in the Oncomine database (Supplementary Figure S1A). The CRISPR function of MCM3 data from the BioGRID ORCS database revealed its function in the cell cycle and potential roles in immune response (Figure 1B). Differential analysis in GSE42656 and GSE50161 showed MCM3 upregulation in malignant samples compared to normal (Figure 1C), adult (Supplementary Figure S1B), and fetal brain tissues (Supplementary Figure S1C, Supplementary Tables S1–S3). Moreover, 255 genes were dysregulated in the two data sets based on the criteria of the absolute logFC >1 and the adjusted *p*-value <0.05 (Figure 1D). The heatmap also showed the differential expression of 255-DEGs (Figure 1E; Supplementary Figures S1D,E). Hub genes analysis revealed the core role of MCM3 in these DEGs (Supplementary Figure S1F) and top 3 ranking among hub genes (Supplementary Figure S1G). Enrichment analysis *via* STRING and ClueGO indicated that DNA replication was the main pathway involved (Figure 1F). The gene effect analysis of the DepMap database also indicated the dependence of cancer cells on MCM3 (Figure 1G). In addition, knockdown of the MCM3 protein level was performed by sh-MCM3 in both D283 and D458 cell lines, which was confirmed by RT-PCR (Figure 1H) and WB (Figure I). The cell viability decreased significantly at 72 h in the sh-MCM3 groups compared to that in the sh-Control groups in both D283 and D458 cell lines. These results revealed the dysregulation of MCM3 in cancers, especially MB, and that MCM3 was essential for the survival of malignant cells in MB.

### MCM3 drives the malignant transformation of non-immune cells *via* DNA replication-related pathways

**FIGURE 2:**
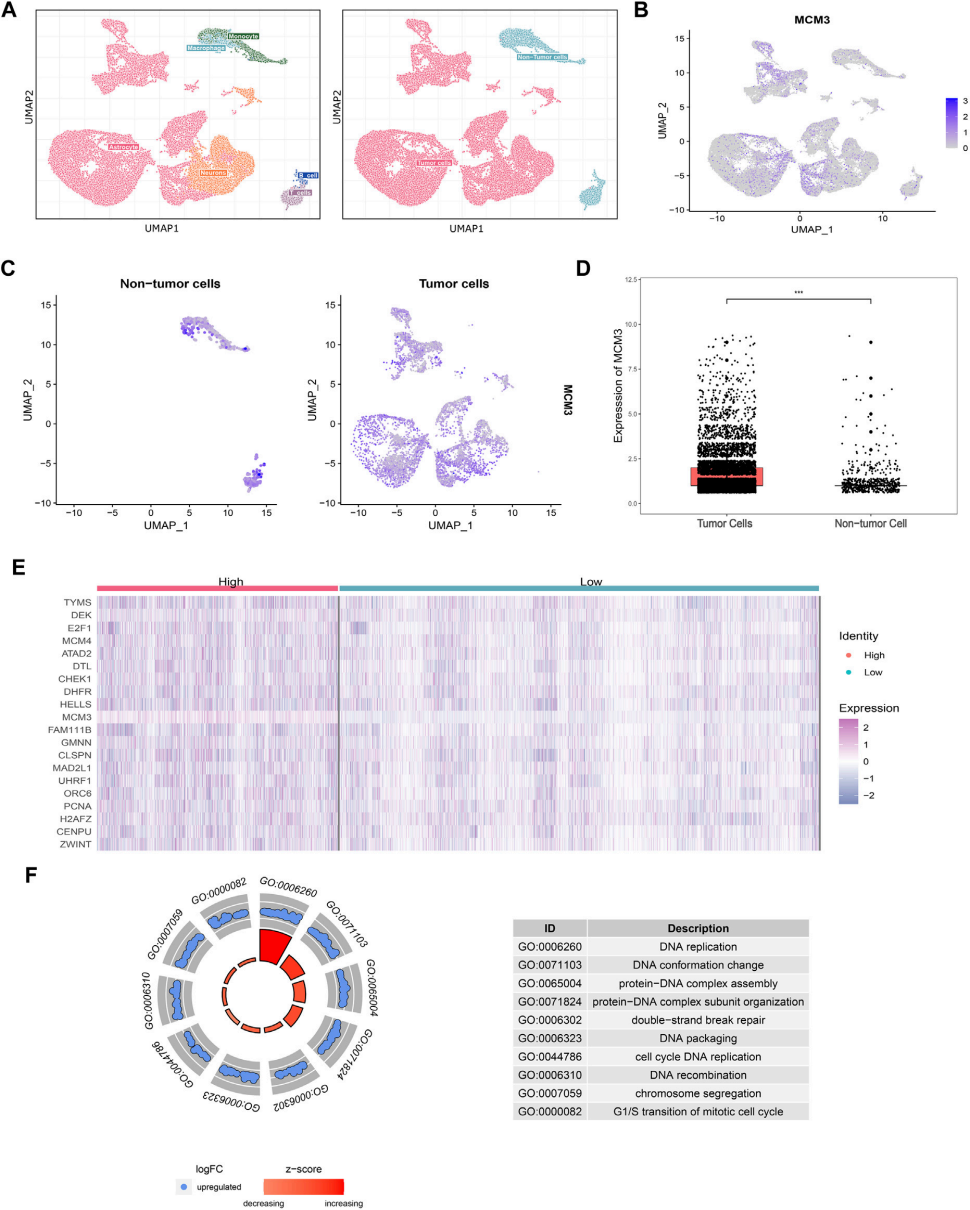
Cell annotations according to SingleR and cell classifications of malignant and non-malignant Cell annotations according to SingleR and cell classifications of malignant and non-malignant cells **(A)**. Heterogeneity of MCM3 expression in different clusters **(B)**. Heterogeneity of MCM3 express ion in tumor and non-tumor cells **(C)**. MCM3 over-expression in tumor cells compared to non-tumor cells **(D)**. Top 20 DEGs **(E)** and the top10 GO **(F)** annotations of 159 DEGs between the two groups of malignant cells (high and low MCM3 expression).

A total of 38,328 cells were divided into 30 clusters comprising six cell types including astrocytes, neurons, B-cells, T-cells, macrophages, and monocytes (Figure 2A) by SingleR. MCM3 expression in all clusters was heterogeneous (Figure 2B). Heterogeneity was also observed in malignant cells, namely non-immune cells, and non-tumor clusters (Figure 2C). Compared to non-tumor cells, MCM3 was significantly overexpressed in tumor cells (Figure 2D). We then investigated the effect of MCM3 on cell biology processes at the single-cell level. A total of 7,536 cells expressing MCM3 were classified as high and low levels according to the mean MCM3 expression level. The top 20 DEGs of the two groups were then identified (Figure 2E). GO analysis was performed of the 159 DEGs after filtering for log fold-change >0.25 and a minimum fraction of 0.25. Like the enrichment analysis in bulk RNA sequencing, the biological processes associated with DNA replication and cell cycle were significantly enriched, in addition to double-strand break repair (Figure 2F). Therefore, MCM3 overexpression might be associated with the malignant transformation of cells *via* the dysregulation of DNA replication and cell cycle.

### MCM3 affects tumorigenesis *via* CDC45-MCM2-7-GINS helicases

**FIGURE 3:**
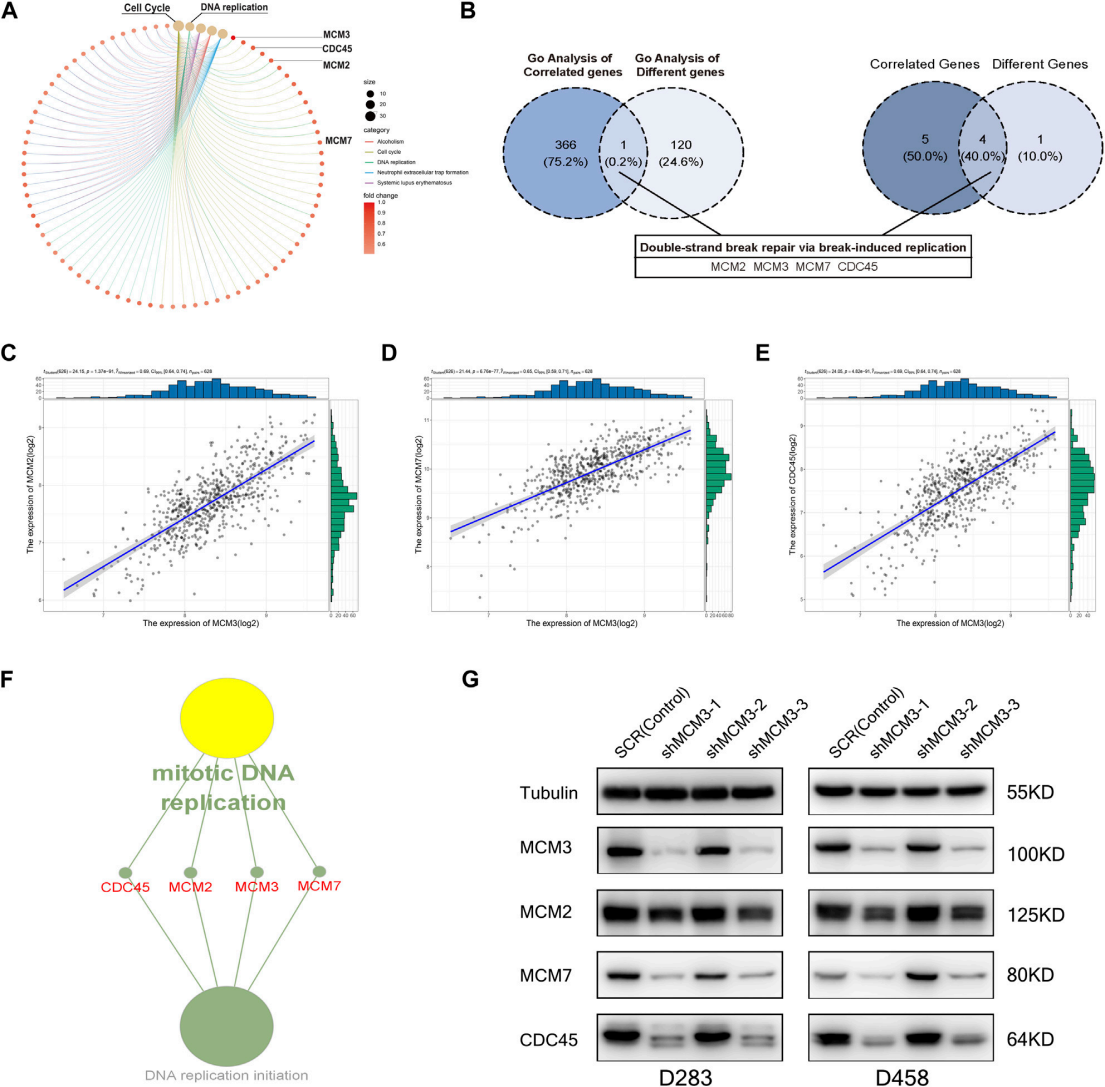
GO enrichment analysis of DEGs for different MCM3 expression levels showing the dysregulation of the cell cycle and DNA replication **(A)**. GO analysis of MCM3-correlated genes and DEGs revealed overlap only for “Double-strand break repair *via* break-induced replication”, with only four genes in this biological process differentially expressed and significantly correlated with MCM3 **(B)**. Significant correlations of MCM3 with MCM2, MCM7, and CDC45 were **(C–E)**. MCM2, MCM3, MCM7, and CDC45 are the main components of the pre-replicative complex **(F)**. MCM7 and CDC45 are correlated with MCM3 at the protein level *via* WB analysis in D283 and D458 cell lines **(G)**.

To investigate the potential mechanism by which MCM3 drives tumorigenesis in MB, we performed GSEA on samples with high and low MCM3expression levels. Dysregulation of MCM3 expression was mainly associated with cell cycle and DNA replication (Figure 3A). Moreover, enrichment analysis of the MCM3-correlated genes and 255 DEGs respectively showed an intersection only for “Double-strand break repair *via* break-induced replication” (Figure 3B). Only four genes—MCM2, MCM3, MCM7, and CDC45—met both the criterion of different expression, and were correlated with MCM3 expression and were included in further analysis (Figures 3C–E). The results of the enrichment analysis demonstrated their important roles in recruitment in the pre-replicative complex (pre-RC) during the initiation of DNA replication (Figure 3F). Furthermore, in both D458 and D283 cells, the protein levels of MCM7 and CDC45 significantly decreased as with MCM3 knockdown (Figure 3G). MCM2 is more stable than other MCMs and might maintain its protein level by reducing cytoplasmic proteolysis, or some other mechanism (Sedlackova et al., 2020). Therefore, MCM3 might play a role in MB via the dysfunction of pre-replicative complex and CDC45-MCM-GINS (CMG) helicases formed by MCM2, MCM3, MCM7, and CDC45.

### MCM3 expression is related to clinical parameters

**FIGURE 4:**
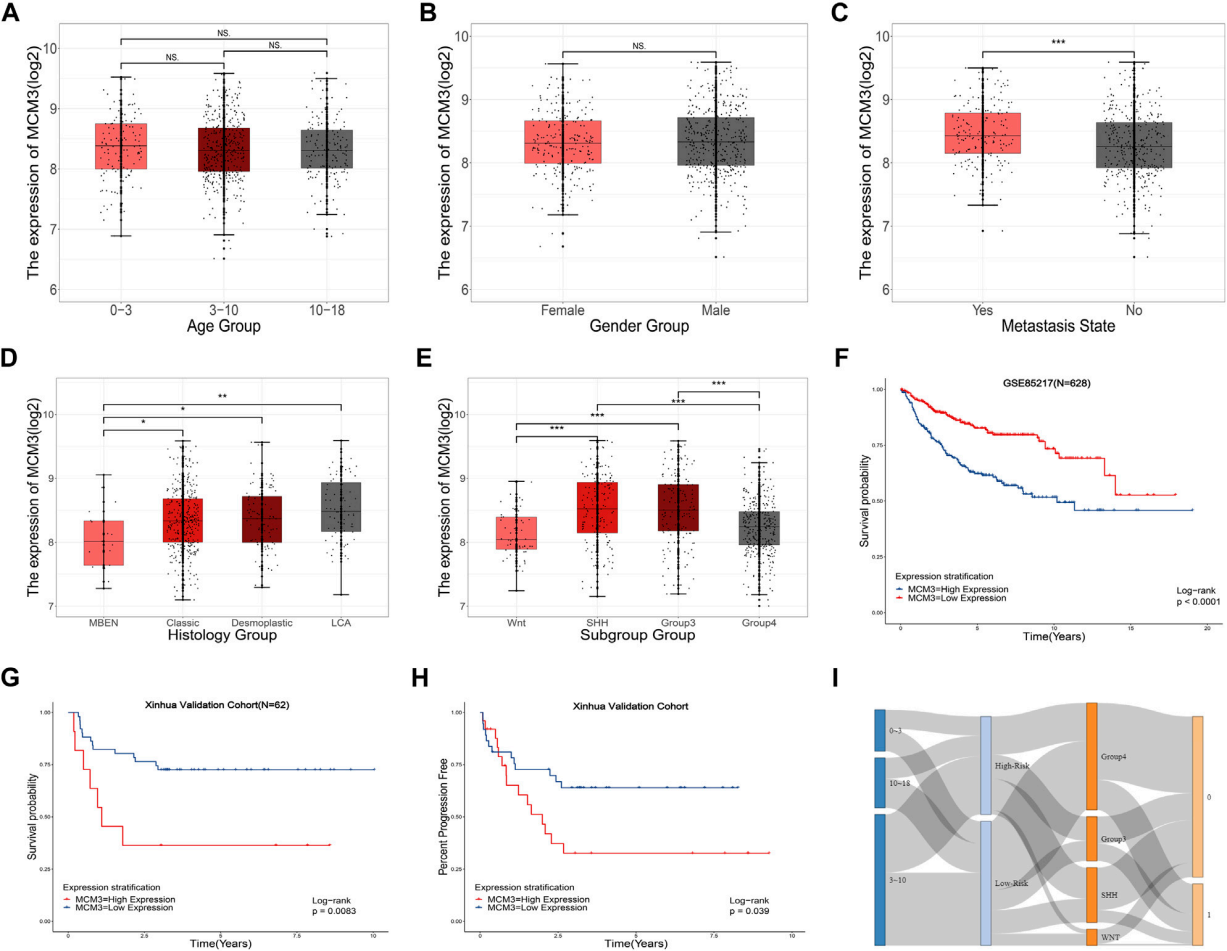
Relationships between MCM3 expression and clinicopathologic features. Correlation of MCM3 expression with age **(A)**, sex **(B)**, metastasis status **(C)**, histology **(D)** and molecular subtypes **(E)**. MCM3 expression is correlated with OS in samples from children (GSE85217, *n* = 628) **(F)**. Validation of the prognostic value of MCM3 in our patient cohort **(G)**. Correlation of MCM3 expression level with metastasis in our cohort **(H)**. Relationships among molecular subtype, MCM3 risk classification, and survival state **(I)**.

Table 1 includes data from 628 patients with prognosis data from GSE85217 and 62 patients with prognosis and RNA-seq data from our cohort. MCM3 expression did not change significantly with age (Figure 4A) or between sexes (Figure 4B); however, children with metastasis showed lower MCM3 expression levels (Figure 4C). Moreover, MCM3 was significantly correlated with histology and molecular subtypes (Figures 4D,E). Thus, more malignant histology and molecular subgroups, such as Large Cell and Anaplastic (LCA), Sonic Hedgehog (SHH)-MB, and Group3-MB, showed higher expression levels of MCM3. In addition, the patients from GSE85217 were classified into high and low-risk groups according to the cut-off value determined by ROC analysis after excluding adult samples. The low-MCM3 group showed better overall survival (Figure 4F), which was validated in our cohort (Figure 4G). Furthermore, our cohort showed that patients with high MCM3 expression had a high risk of metastasis (Figure 4H). In addition, the Sankey plots also revealed higher proportions of low-risk patients in WNT and Group4 MB, and better prognosis in the low-risk group (Figure 3I). Therefore, MCM3 expression was related to current major clinical parameters and had potential clinical significance.

**TABLE 1:** Data of RNA-seq included for Kaplan-Meier analysis.

	GSE85217	Our cohort
	(*n* = 628)	(*n* = 62)
Gender		
Female	212 (33.8%)	25 (40.3%)
Male	405 (64.5%)	37 (59.7%)
NA	11 (1.8%)	NA
Age		
Mean (SD)	7.30 (4.10)	5.20 (3.22)
Median (Min, Max)	7.00 (0.24,17.3)	4.45 (0.334,16.1)
Histology		
Classic	340 (54.1%)	41 (66.1%)
Desmoplastic	86 (13.7%)	7 (11.3%)
LCA	67 (10.7%)	9 (14.5%)
MBEN	17 (2.7%)	5 (8.1%)
NA	118 (18.8%)	NA
Metastasis		
Yes	165 (26.3%)	28 (45.2%)
No	333 (53.0%)	34 (54.8%)
NA	130 (20.7%)	NA
Dead		
Yes	147 (23.4%)	21 (33.9%)
No	398 (63.4%)	41 (66.1%)
NA	83 (13.2%)	NA
Overall Survival (years)		
Mean (SD)	4.88 (3.66)	3.93 (2.74)
Median (Min, Max)	3.92 (0.0192,19.0)	3.29 (0.186,10.0)
NA	90 (14.3)	NA

### MCM3 dysregulation remodels the immune microenvironment and affects multiple cell death-related processes

**FIGURE 5:**
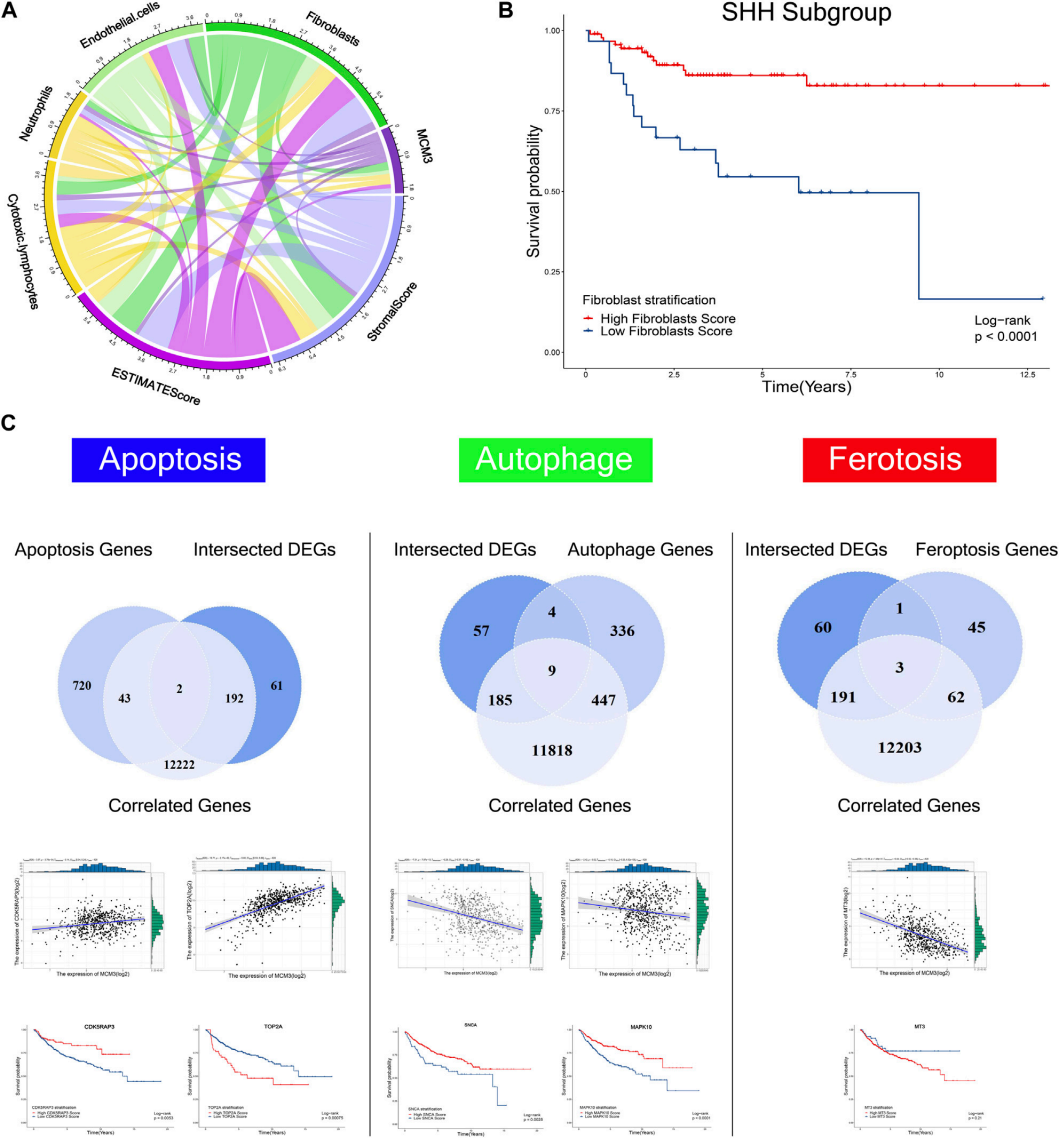
Correlations of MCM3 with fibroblasts, endothelial cells, neutrophils, cytotoxic lymphocytes, estimate score, and stromal score **(A)**. Association of fibroblasts with OS in the SHH subtype **(B)**. Kaplan–Meier analysis of genes related to apoptosis, autophagy, and ferroptosis that were correlated with MCM3 and differentially expressed. CDK5RAP3, TOP2A, SNCA, MAPK10 are correlated with MCM3 and associated with OS **(C)**.

To systematically investigate the effect of MCM3 dysregulation in MB, we first analyzed tumor-infiltrating cells via three algorithms, including ESTIMATE, CIBERSORT, and XCELL. The results revealed the differential infiltration of immune cells, such as cytotoxic lymphocytes, fibroblasts, CD4 T cells, macrophages, etc. (Supplementary Figure S2). MCM3 was correlated with varied components of the immune microenvironment based on the ESTIMATE score (Figure 5A). Subgroup analysis of four molecular subgroups revealed that the varied immune components were associated with OS in different subtypes, such as fibroblasts in SHH (Figure 5B), neutrophils and stromal score in SHH (Supplementary Figures S3A,B), neutrophils in WNT MB (Supplementary Figure S3C), and stromal and estimate scores in Group4 MB(Supplementary Figures S3D,E). In addition, other biological processes were also analyzed, including apoptosis, autophagy, and ferroptosis (Figure 5C). First, the DEGs and MCM3-correlated genes were analyzed with the three process-related genes to obtain the intersection genes; for example, CDK5RAP3 and TOP2A in apoptosis, DNM3, GABARAPL1, GABBR2, OPTN (Supplementary Figures S3F–I), SCNA and MAPK10 in autophagy (Figure 5C), and MT3 in ferroptosis (Figure 5C). Only DNM3, GABARAPL1, GABBR2, SNCA (Supplementary Figures S3J -L), CDK5RAP3, TOP2A, SCNA and MAPK10 (Figure 5C) were associated with OS. Therefore, MCM3 might affect the development of MB *via* these genes.

### Abnormal MCM3 demethylation may contribute to its expression dysregulation

**FIGURE 6:**
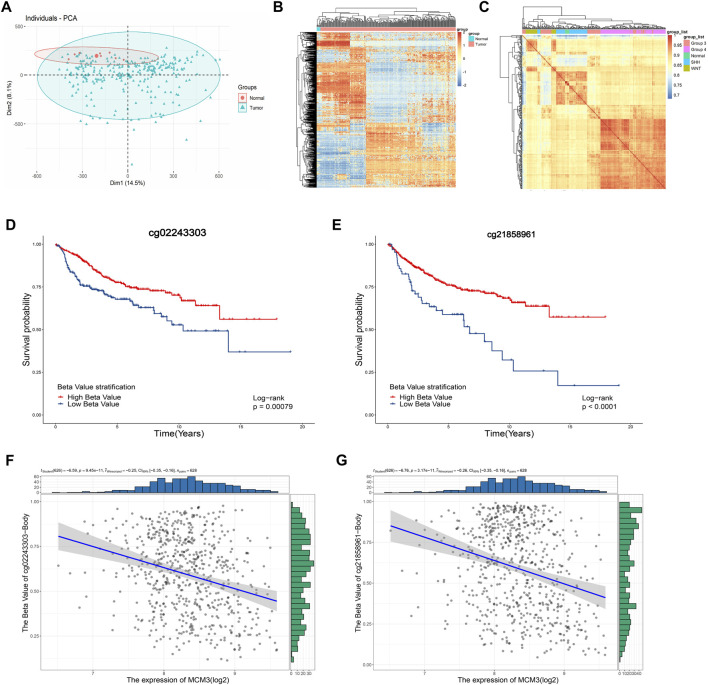
The PCA and heatmap showing the significant difference between normal and tumor samples **(A,B)** and the high correlation within groups **(C)**. The “cg02243303” and “cg2185896” probes are significantly associated with OS **(D,E)** and negatively correlated with MCM3 expression **(F,G)**.

**FIGURE 7:**
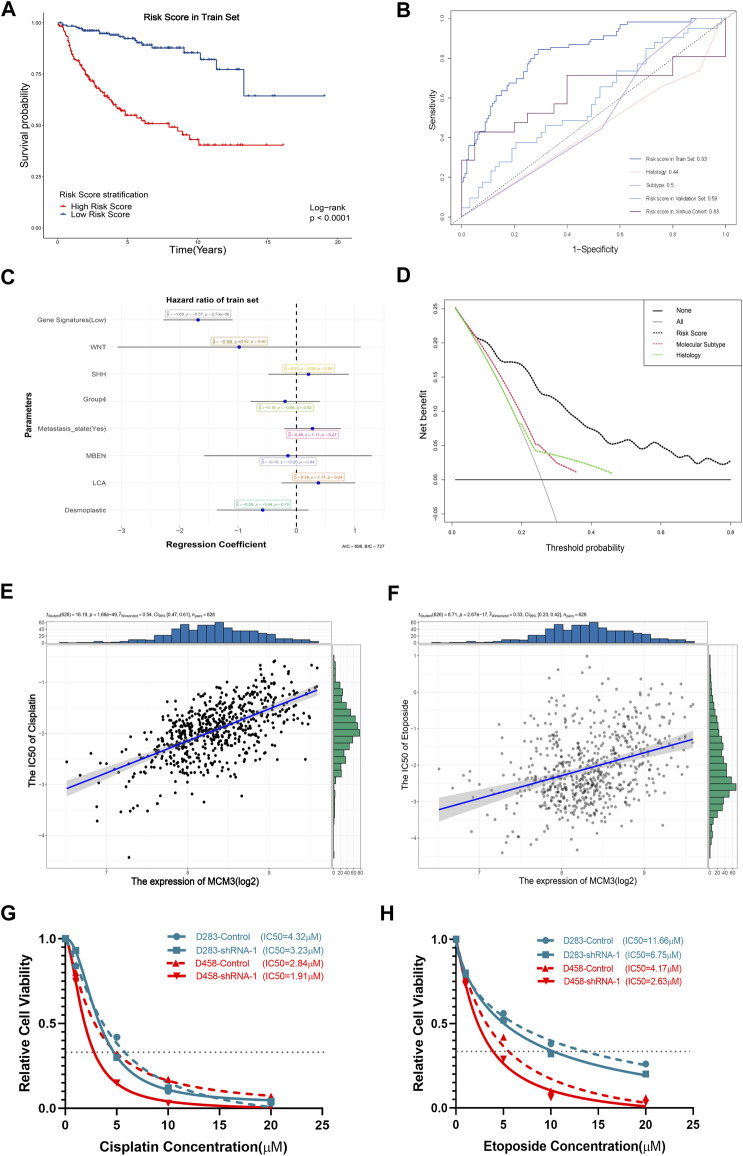
Significant association of the gene signature based on MCM3-related genes with OS **(A)**. The AUC values of the training set, internal validation set, and our cohort are higher than those for histological classification and molecular subtyping **(B)**. The forest plot showed that the gene signature was the only independent factor **(C)**. DCA demonstrating the better performance of the risk score compared to the current histological classification and molecular subtyping **(D)**. Positive expression of MCM3 expression with sensitivity to etoposide **(E)** and cisplatin **(F)**. Decreased IC_50_ values of cisplatin **(G)** and etoposide **(H)** in the sh-MCM3 groups in both D283 and D458 cell lines.

Considering the epigenetic disorders in pediatric brain tumors (Lin et al., 2016; Northcott et al., 2017; Petralia et al., 2020), we investigated the cause of MCM3 dysregulation at the methylation level. The PCA and heatmap revealed significant differences between the tumor and normal groups (Figure 6A), while the heatmaps demonstrated the acceptable quality control (Figures 6B–C). MCM3 probes were then obtained according to the beadchip annotation. Among the 14 probes for MCM3, probes 7 and 13 were filtered, respectively, after data normalization of GSE85212 and GSE54880. “cg02243303” and “cg21858961” differed significantly between normal and tumor groups and were associated with OS (Figures 6D–E). Moreover, MCM3 expression was correlated with the methylation level of the two sites (Figures 7F–G). Therefore, “cg02243303” and “cg21858961” hypomethylation might be involved in MCM3 regulation in MB.

### The prognostic model based on MCM3-related genes performs better than current tumor classifications

We next investigated the clinical significance of MCM3. First, given its extensive influence, genes correlated with MCM3 (11,008 genes) and associated with OS (RAP2B, ARHGEF40, ADGRG6, ALS2CL, FZD4, TJP2, EIF2AK3, FBLIM1, DGLUCY, C6orf141, APLN, FCRL1, ZCCHC13, ZMYND15, FAM163B, LBHD1, UEVLD) were included in univariate and lasso regression analyses to construct gene signatures (Supplementary Figures S4A,B). Kaplan–Meier analysis revealed that a low risk score showed a dramatically longer OS compared to that for a high risk score (Figure 7A). The testing data set for internal validation (Supplementary Figure S4C) and our cohort data (Supplementary Figure S4D) also confirmed the prognostic value of the gene signature. The AUC of 5-year survival was 0.83 (Figure 7B). Although the AUC values of the validation set and our cohort were relatively lower, all were significantly higher than those of histological and molecular subtypes (Figure 7B). Moreover, the multivariate Cox regression analysis revealed that only the risk score was the independent factor (Figure 7C). In addition, the nomogram model predicting 1-year and 5-year probabilities can be explored on the website (https://cll12345.shinyapps.io/DynNomapp/). The C-index of the nomogram was 0.784 (CI: 0.723–0.831), suggesting its reliability. Moreover, decision curve analysis (DCA) of the nomogram showed that the prognostic model performed better than the current strategies for MB classification (Figure 7D). Finally, owing to the predominance of conventional chemotherapies in the treatment of MB, we evaluated the correlation between MCM3 expression and drug sensitivities. Patients with low MCM3 expression were more sensitive to etoposide and cisplatin treatment (Figures 7E,F). Furthermore, we validated the decrease in the IC50 of etoposide and cisplatin by knocking down MCM3 expression in both D283 and D458 cell lines (Figures 7G,H). Therefore, MCM3 might be used to guide prognostic assessment and MCM3 targeted therapy might be a new potential strategy to reduce chemotherapy doses, which is of great significance for individualized chemotherapy in children.

## Discussion

The results of this study demonstrated the dysregulation of MCM3 in most common cancers and showed its expression level in MB cell lines of pediatric brain tumors, which indicated its potential correlation with tumorigenesis. The CRISPR screening data, covering multiple cancer cell lines from the BioGRID ORCS database, also indicated its essential role in malignant cells and potential relationships with apoptosis, autophagy, and ferroptosis. We then showed MCM3 overexpression in MB, its core place in DEGs, and the biological process it drove at bulk and single-cell RNA-seq levels. We also investigated the correlations between MCM3 and clinical features, finding that the MCM3 expression level was related to high-risk clinicopathologic and molecular subtypes and poor prognosis. We further performed GSEA based on high and low MCM3 expression levels and found that MCM3 might promote tumorigenesis through the dysfunction of the pre-replicative complex and CDC45-MCM-GINS (CMG) helicases formed by MCM2, MCM3, MCM7, and CDC45. Considering the reported relationships with immune response, apoptosis, autophagy, and ferroptosis, we also found that MCM3 was correlated with immune microenvironment components and might affect genes related to the above biological processes, such as CDK5RAP3, TOP2A, OPTN, MAPK10, etc., which indicated that MCM3 might affect tumorigenesis through a variety of mechanisms. In addition, we investigated the mechanisms of MCM3 dysregulation at the DNA methylation level and identified two differential sites that were associated with OS and correlated with MCM3 expression, which indicated that abnormal methylation might result in MCM3 dysregulation. Finally, we discovered the correlation of MCM3 expression with sensitivity to chemotherapy medications, including etoposide and cisplatin.

MCM3, a member of the MCM family of DNA-dependent ATPases that bind to replication origins and support a single round of DNA replication, has demonstrated dysfunction in most cancers. As shown in Figure 1, MCM3 is upregulated in various tumors compared to normal tissues. MB is a highly heterogeneous tumor with the highest incidence and malignancy. Molecular subtypes have been described as a reference for prognosis and individual therapy; however, the high costs of this analysis limit its popularity in primary medical care. Thus, there is an urgent need to identify low-cost biomarkers to guide clinical decision-making. As reported in other tumors, MCM3 is also upregulated in MB based on bulk RNA-seq data. Moreover, we also investigated its expression at the single-cell level. As shown in Figure 2, MCM3 was overexpressed in tumor cells and might be related to malignant transformation.

The present study also evaluated the impact of MCM3 dysregulation according to previous reports (Söling et al., 2005; Puustinen et al., 2020). First, we studied the association of MCM3 expression within the MB microenvironment via different methods. We found the differential immune infiltration between high and low MCM3 expression levels, including higher infiltration levels of cytotoxic lymphocytes in the high MCM3 group and higher infiltration levels of fibroblasts and M2 macrophages in the low MCM3 group. Moreover, immune components such as neutrophils and fibroblasts, as well as stromal and estimate scores, were associated with OS in MB subtypes, indicating the potential role of MCM3 by affecting immune infiltration. We also evaluated the association of MCM3 with apoptosis, autophagy, and ferroptosis-related genes, in which some differentially expressed genes were correlated with MCM3 and associated with OS. Therefore, MCM3 might also influence prognosis via these genes, which requires further robust experimental verification.

Considering the low mutation burden of pediatric brain tumors, we investigated the mechanism of MCM3 expression dysregulation. As expected, 2 of 13 sites in MCM3 showed hypermethylation in MB and were associated with OS. Moreover, the hypermethylation state was significantly correlated with MCM3overexpression. Previous studies also reported that MCM3 hypomethylation could increase its expression in hepatocellular carcinoma (Hua et al., 2020) and osteosarcoma (Zhou et al., 2021), and was negatively associated with prognosis. We also identified that “double-strand break repair *via* break-induced replication” might be the most affected biological processes in GSEA of MCM3 correlated and differential genes between the high and low MCM3 expression groups (Figure 3B). Meanwhile, MCM7 and CDC45, which were involved in this process, were correlated with MCM3 and differentially expressed between normal and tumor tissues.

Our results also verified that MCM3 expression was correlated with other clinical parameters, especially molecular subtypes and OS, with low-risk classification based on MCM3 expression commonly observed in the WNT MB and surviving groups (Figure 4). This indicated that MCM3 might be a low-cost biomarker for MB risk classification. MCM3 could also guide for chemotherapy selection according to its robust correlation (*r* ^2^ = 0.54 and *r* ^2^ = 0.33) with cisplatin and etoposide, which might contribute to individualized therapy to reduce their toxic side effects in children (Figures 7E,F).

Despite the results, this study has several limitations. First, while our results demonstrated the correlations between MCM3 and multiple biological processes through bioinformatics analysis, further validation studies are needed to reveal the crosstalk between them. Second, further studies of the MCM-associated mechanisms via CMG helicases might also provide a direction for novel target development. In addition, although our prognostic model demonstrated a better performance than current histological and molecular subtypes, further clinical translation is needed.

## Conclusion

Overall, we systematically studied abnormal MCM3 expression and affected biological processes in bulk and single-cell RNA-seq. Our results showed MCM3 overexpression in MB and its relationships to clinical parameters. We also discovered that MCM3 might affect varied biological processes by aberrant methylation of MCM3 and dysregulation of the complex consisting of MCM2, MCM3, MCM7, and CDC45. Importantly, our results indicated that MCM3 might serve as a potential biomarker of prognosis prediction and better guidance compared to current histological and molecular classification systems for clinical decision-making.

## Data Availability

The datasets presented in this study can be found in online repositories. The names of the repository/repositories and accession number(s) can be found in the article/Supplementary Material.

## Supplementary material

SUPPLEMENTARY FIGURE S1 Dysregulation of MCM3 in most cancers compared to corresponding normal tissues . MCM3 upregulation in the tumor group compared to adult normal samples from GSE42656 **(B)**. MCM3 upregulation in the tumor group compared to fetal normal samples from GSE42656 **(C)**. The adult normal and medulloblastoma samples from GSE42656 clustered according to the expression of 255-DEGs **(D)**. The fetal normal and medulloblastoma samples from GSE42656 clustered respectively according to the expression of 255-DEGs **(E)**. The core position of MCM3 among DEG interactions **(F)**. Ranking of MCM3 within the top three hub genes **(G)**.

SUPPLEMENTARY FIGURE S2 Estimation of tumor-infiltrating cells via three algorithms, including ESTIMATE, CIBERSORT, and XCELL, showing differential infiltration of immune cells, including cytotoxic lymphocytes, fibroblasts, CD4 T cells, macrophages, etc.

SUPPLEMENTARY FIGURE S3 Association of neutrophils with OS in the SHH subtype . Association of stromal score with OS in the SHH subtype **(B)**. Association of neutrophils with OS in WNT MB **(C)**. Association of stromal score with OS in the Group4 subtype **(D)**. Associations of estimate score with OS in Group4 MB **(E)**. Negative correlations of the expression of autophagy-related genes (DNM3, GABARAPL1 GABBR2, and OPTN) with MCM3 expression **(F–I)**. Relationship of the expression of autophagy-related genes (DNM3, GABBR2 and GABARAPL1) with OS **(J–L)**.

SUPPLEMENTARY FIGURE S4 Lasso regression analysis with MCM3-correlated genes in GSE85217, in which 17 genes constituted the multi-gene signature . Association of the risk score with OS in the internal validation set **(C)**. Association of the risk score with OS in our cohort **(D)**.
