# Corrigendum: Systematic analysis of MCM3 in pediatric medulloblastoma *via* multi-omics analysis

**DOI:** 10.3389/fmolb.2022.1076243

**Published:** 2022-11-14

**Authors:** Liangliang Cao, Yang Zhao, Zhuangzhuang Liang, Jian Yang, Jiajia Wang, Shuaiwei Tian, Qinhua Wang, Baocheng Wang, Heng Zhao, Feng Jiang, Jie Ma

**Affiliations:** Department of Pediatric Neurosurgery, Xin Hua Hospital Affiliated to Shanghai Jiao Tong University School of Medicine, Shanghai, China

**Keywords:** MCM3, medulloblastoma, multi-omics, DNA methylation, single cell, RNA sequencing, prognosis, nomogram

In the published article, the following **Authors** names “Zhuangzhuag Liang” and “Shuangwei Tian”, should be written as “Zhuangzhuang Liang” and “Shuaiwei Tian”.

In the published article, there was an error in the **Author** list, and the **Equal Contribution** dagger for author “Liangliang Cao” was erroneously missed.

The authors apologize for this error and state that this does not change the scientific conclusions of the article in any way. The original article has been updated.

