# Supplementary figures and images for: Systematic analysis of MCM3 in pediatric medulloblastoma via multi-omics analysis
Source: Front Mol Biosci. 2022 Sep 5;9:815260. doi: 10.3389/fmolb.2022.815260 (PMC9483186; doi:10.3389/fmolb.2022.815260)

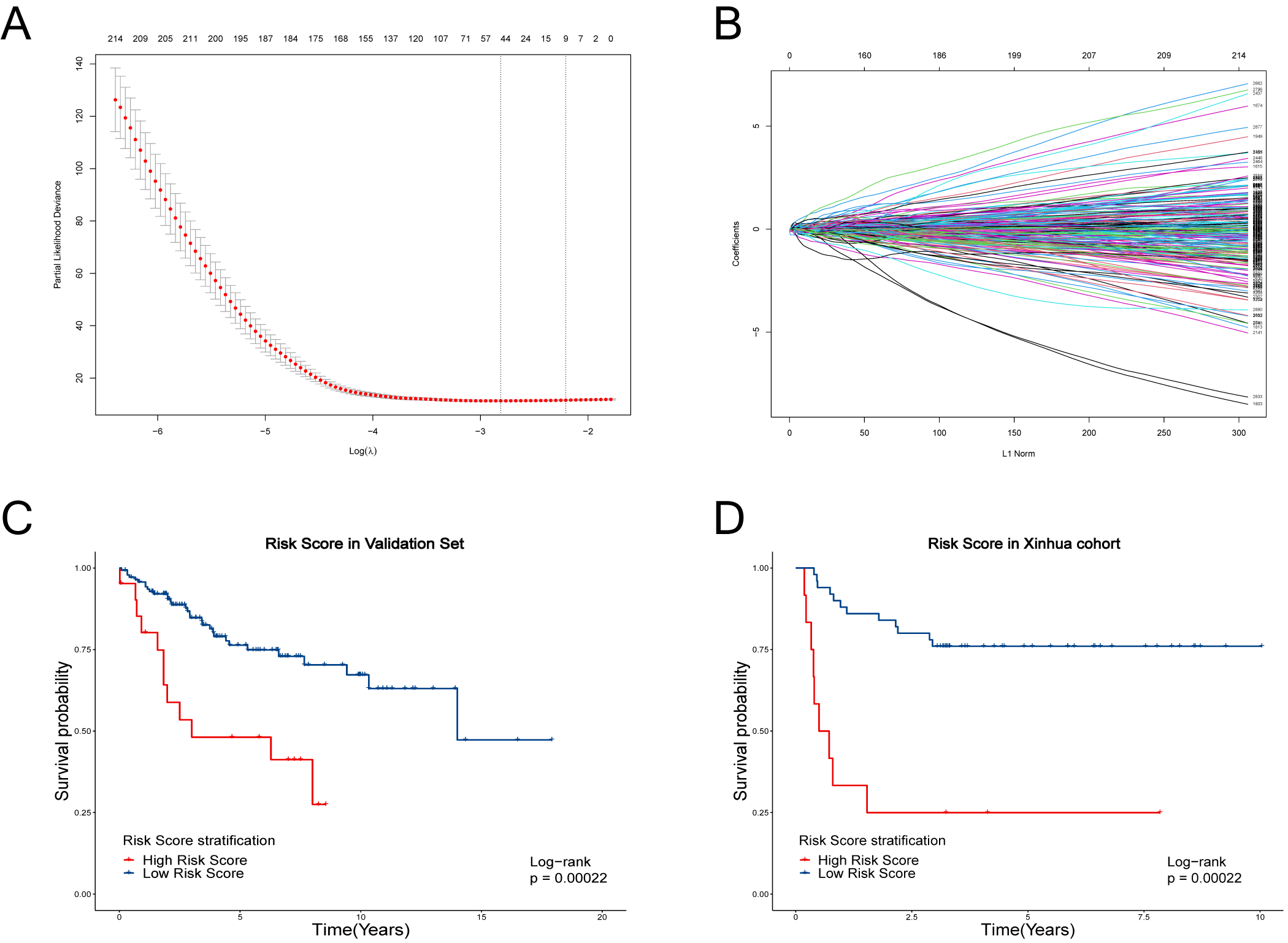

Supplement: Supplementary file 4 [file Image4.TIF]

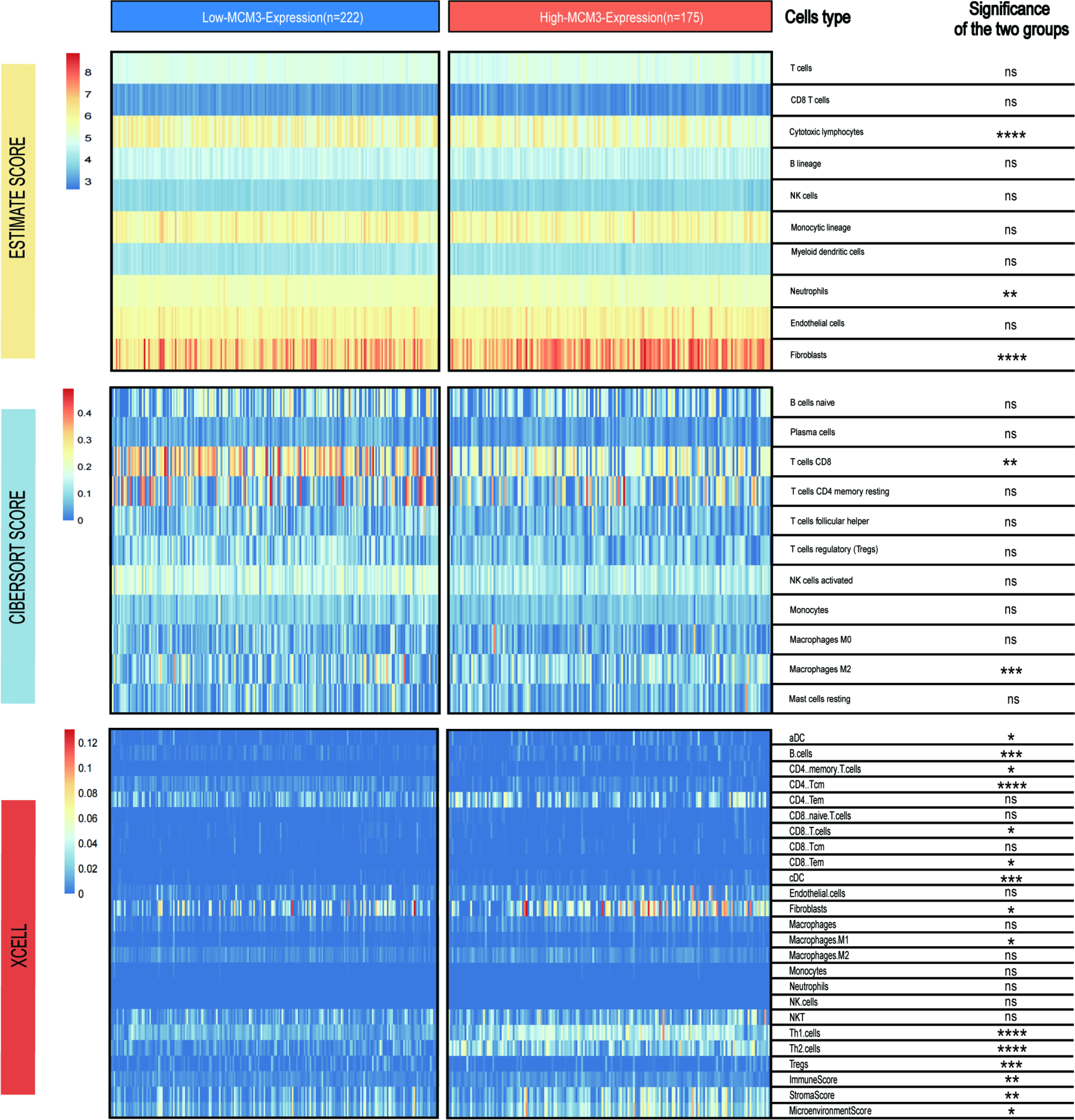

Supplement: Supplementary file 5 [file Image2.TIF]

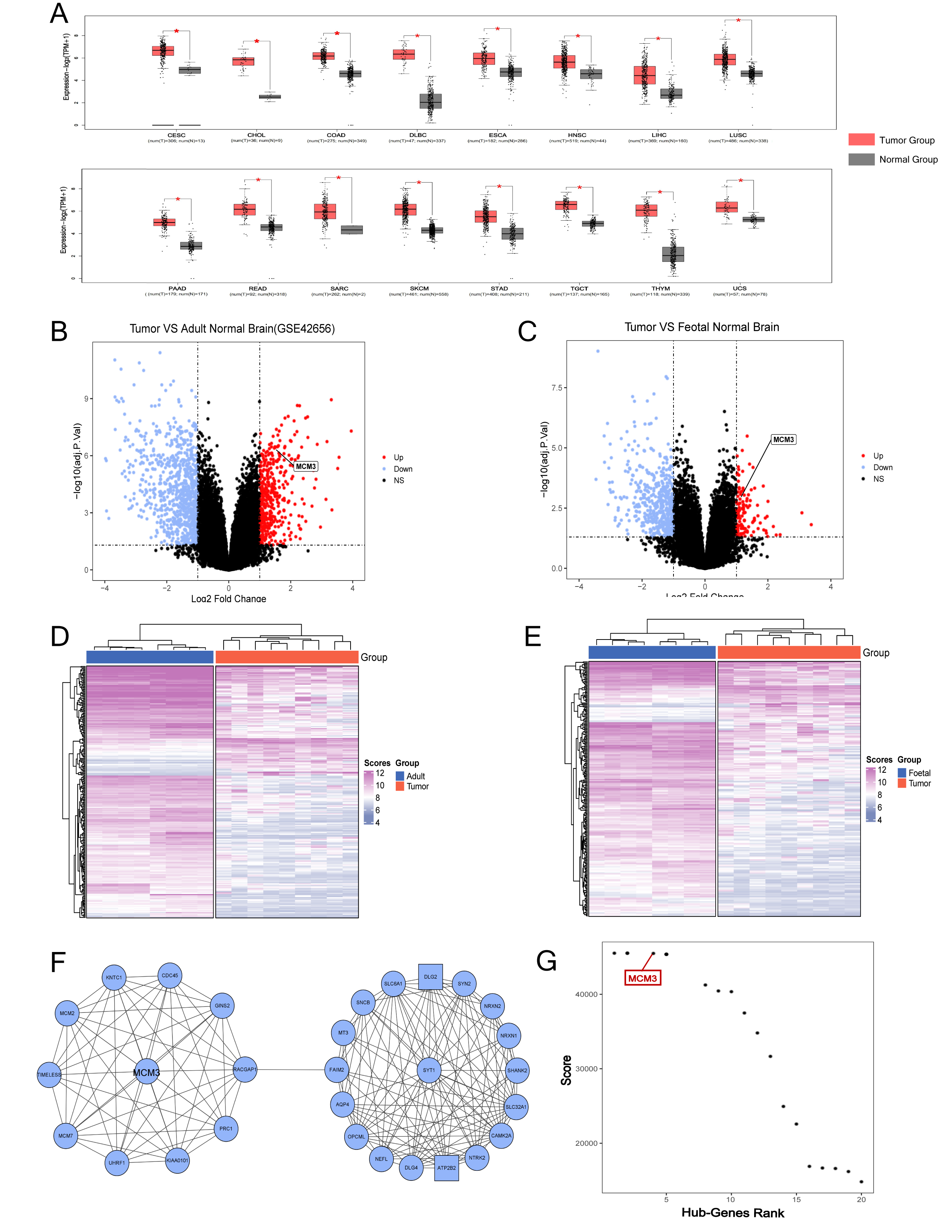

Supplement: Supplementary file 6 [file Image1.TIF]
